# Fenotipo hipertensión-obesidad abdominal como indicador de disglucemia y resistencia a la insulina

**DOI:** 10.15446/rsap.V25n6.110831

**Published:** 2023-11-01

**Authors:** Eduardo Cabrera-Rode, Brayam Javier Loaiza-Romero, Janet Rodríguez-Acosta, Ileana Cubas-Dueñas, José Hernández-Rodríguez, Oscar Díaz-Díaz

**Affiliations:** 1 EC: Biol. Ph. D. Ciencias Biológicas, Universidad de la Habana. Instituto de Endocrinología (INEN), Universidad de Ciencias Médicas de la Habana. La Habana, Cuba. eduardo.cabrerarode@gmail.com Instituto de Endocrinología (INEN) Universidad de Ciencias Médicas de la Habana La Habana Cuba; 2 BL: MD. Esp. Endocrinología. Instituto de Endocrinología (INEN), Universidad de Ciencias Médicas de la Habana. La Habana, Cuba. brayamloaiza@gmail.com Universidad de Ciencias Médicas de la Habana La Habana Cuba; 3 JR: Tecnol. Salud. Esp. Laboratorio Clínico. Instituto de Endocrinología (INEN), Universidad de Ciencias Médicas de la Habana. La Habana, Cuba. yanetra@infomed.sld.cu Universidad de Ciencias Médicas de la Habana La Habana Cuba yanetra@infomed.sld.cu; 4 IC: MD. Esp. Inmunología. Instituto de Endocrinología (INEN), Universidad de Ciencias Médicas de la Habana. La Habana, Cuba. ileana.cubas77@gmail.com Universidad de Ciencias Médicas de la Habana La Habana Cuba; 5 JH: MD. Esp. Endocrinología. Instituto de Endocrinología (INEN), Universidad de Ciencias Médicas de la Habana. La Habana, Cuba. pepehdez@infomed.sld.cu Universidad de Ciencias Médicas de la Habana La Habana Cuba; 6 OD: MD. Esp. Endocrinología. Instituto de Endocrinología (INEN), Universidad de Ciencias Médicas de la Habana. La Habana, Cuba. diazdiaz@infomed.sld.cu Universidad de Ciencias Médicas de la Habana La Habana Cuba

**Keywords:** Obesidad abdominal, hipertensión, disglucemia, diabetes mellitus, prediabetes, resistencia a la insulina *(fuente: DeCS, BIREME)*, Abdominal obesity, hypertension, dysglycaemia, diabetes mellitus, prediabetes, insulin resistance *(source: MeSH, NLM)*

## Abstract

**Objetivo:**

Determinar la utilidad del fenotipo hipertensión-obesidad abdominal como indicador de disglucemia y resistencia a la insulina.

**Material y Métodos:**

Estudio descriptivo transversal con 964 personas adultas (449 mujeres y 515 hombres), que asistieron al Instituto de Endocrinología con riesgo de *diabetes mellitus.* Se analizaron variables demográficas (edad, sexo, color de la piel), clínicas (tensión arterial y Acantosis nigricans), antropométricas (peso, talla, circunferencia de la cintura e índice de masa corporal) y de laboratorio (glucemia basal y estimulada con la prueba de tolerancia a la glucosa oral e insulinemia). Se calculó el índice de resistencia a la insulina. El fenotipo hipertensión-obesidad abdominal se definió como la presencia de presión sistólica ≥130 mm Hg o presión diastólica ≥80 mm Hg o hipertensión arterial tratada, circunferencia de cintura ≥80 cm en mujeres y ≥90 cm en hombres. Se calculó la sensibilidad, la especificidad y los valores predictivos del fenotipo hipertensión obesidad abdominal para identificar disglucemia y resistencia a la insulina.

**Resultados:**

Los individuos con el fenotipo hipertensión-obesidad abdominal mostraron mayor proporción de alteraciones del metabolismo de la glucosa y de resistencia a la insulina que las personas sin el fenotipo (p<0,0001). El fenotipo hipertensión-obesidad abdominal identifica mejor a personas con presencia de prediabetes doble, *diabetes mellitus* y resistencia a la insulina, pues muestran sensibilidades altas (85,9%, 77,5%, y 68,9%, respectivamente) y valores predictivos negativos altos (97,9%, 95,8%, y 74,0%, respectivamente).

**Conclusiones:**

El fenotipo hipertensión-obesidad abdominal es una opción sencilla, útil para identificar a personas con disglucemia y resistencia a la insulina.

## Abstract (en)

**Objective:**

To determine the usefulness of the hypertension-abdominal obesity phenotype as an indicator of dysglycaemia and insulin resistance.

**Materials and Methods:**

Cross-sectional descriptive study of 964 adults (449 women and 515 men) who attended the Institute of Endocrinology with risk of *diabetes melli*tus. Demographic (age, sex, skin colour), clinical (blood pressure and acanthosis nigricans), anthropometric (weight, height, waist circumference and body mass index) and laboratory (basal and oral glucose tolerance test-stimulated blood glucose and insulinaemia) variables were analysed. The insulin resistance index was calculated. The abdominal hypertension-obesity phenotype was defined as the presence of systolic pressure ≥130 mm Hg and/or diastolic pressure ≥80 mm Hg or treated hypertension, waist circumference ≥80 cm in women and ≥90 cm in men. Sensitivity, specificity and predictive values of the abdominal obesity hypertension phenotype were calculated to identify dysglycaemia and insulin resistance.

**Results:**

Individuals with the hypertension-abdominal obesity phenotype showed a higher proportion of impaired glucose metabolism and insulin resistance than those without the phenotype (p<0.0001). The hypertension-abdominal obesity phenotype better identifies persons with the presence of double prediabetes, *diabetes mellitus* and insulin resistance, as they show high sensitivities (85.9%, 77.5%, and 68.9%, respectively), and high negatives predictive values (97.9%, 95.8%, and 74.0%, respectively).

**Conclusions:**

The hypertension-abdominal obesity phenotype is a simple, useful option for identifying persons with dysglycaemia, and insulin resistance.

La obesidad contribuye al desarrollo de múltiples enfermedades, entre las cuales se describen: la *diabetes mellitus* (DM) tipo 2 (DM2), las dislipidemias (DLP), la enfermedad de las arterias coronarias y cerebrales, así como la hipertensión arterial (HTA). Asimismo, se ve asociada a comorbilidades no metabólicas, entre las que se citan: la enfermedad por reflujo gastroesofágico, varios tipos de cáncer, incluidos las neoplasias de próstata, colon, mama, útero, esófago, el carcinoma hepatocelular, asma, enfermedades del hígado y de vesícula biliar, problemas pulmonares, enfermedad renal, osteoartritis, problemas reproductivos, muerte prematura, trastornos del sueño, depresión y algunas enfermedades de tipo musculoesquelético 1. Como tal, la obesidad general es la quinta causa de enfermedades no transmisibles 2,3.

Si bien el índice de masa corporal (IMC) es uno de los métodos más utilizados para medir la obesidad, ha sido criticado por ser una medida de la obesidad general y puede no representar con precisión la localización de la adiposidad. Se sugiere que la adiposidad visceral abdominal es la medida más importante de la obesidad 2,4.

Wang *et al.*^5^ asociaron el aumento de la circunferencia de la cintura (CC) con la presión arterial elevada, incluso en ausencia de ganancia de peso. Los autores 5 sugieren que el manejo de la tensión arterial (TA), teniendo en cuenta solo el aumento del IMC, podría pasar por alto a las personas con riesgo de HTA con obesidad abdominal, pero sin exceso de peso. Otros investigadores 6,7 refieren que la mortalidad por enfermedad coronaria es alta en personas con IMC normal y obesidad abdominal. En líneas generales, se cree que la obesidad abdominal -evaluada mediante la CC oel índice cintura/cadera (ICC)- es mejor predictor de riesgo cardiometabólico que la obesidad generalizada, evaluada por el IMC 8.

Los pacientes con obesidad abdominal tienen otros factores de riesgo cardiovascular (RCV) -además de los ya descritos- que incrementan su riesgo global. De ahí la importancia de determinar los factores de riesgo asociados con dicho estado, para poder implementar -de manera precoz- medidas preventivas, en una etapa en que las acciones de salud son más efectivas 9.

La medición de la circunferencia de la cintura es fácil de realizar, efectiva y económica para detectar obesidad abdominal; esta última se asocia con la HTA y otras alteraciones metabólicas 5,7-9. El fenotipo hipertrigliceridemia-cintura abdominal alterada ha sido el binomio más estudiado y mejor caracterizado. Se ha reafirmado al fenotipo hipertrigliceridemia-cintura abdominal alterada como predictor de RCV, mediante su asociación con alteraciones metabólicas, entre las que figuran: otras alteraciones del perfil lipídico, la HTA, la DM2 y la resistencia a la insulina (RI). El fenotipo hipertrigliceridemia-cintura abdominal alterada también se ha reconocido como una herramienta que apoya la detección sencilla y menos costosa de los trastornos antes mencionados 10-12.

Para simplificar aún más la identificación de personas con riesgo cardiometabólico, se ha propuesto realizar la medición de la CC y la TA, dos parámetros clínicos de fácil y rápida evaluación, que no requieren mediciones bioquímicas 13. En Cuba se llevó a cabo un estudio en el cual se investigaron varios fenotipos en personas con exceso de peso, con el objetivo de determinar la presencia del llamado "síndrome metabólico" 14. En dicha investigación se analizó la combinación de varios binomios de sus componentes y se encontró que el fenotipo hipertensión-obesidad abdominal (FHTOA) fue la combinación más frecuente en personas con exceso de peso 14. Asimismo, Parlá *et al.*^15^ encontraron que el FHTOA es el mejor binomio para identificar a personas con riesgo cardiovascular moderado-alto según criterios de *Gaziano,* lo cual apoya la importancia del empleo de este fenotipo.

Varios grupos de investigadores han encontrado que las personas con obesidad abdominal o HTA, de manera independiente, se asocian con un incremento del riesgo a desarrollar RI, prediabetes y DM 8,16-20. Por tanto, suponemos que la combinación de estos componentes para la conformación del FHTOA podría también ser útil en la detección de sujetos con disglucemia y RI.

Cabrera-Rode *et al.*^14^ encontraron que las personas con FHTOA mostraban una frecuencia superior de glucemia alterada en ayunas (GAA), en comparación con aquellos sin el fenotipo. Por consiguiente, los investigadores revelan el FHTOA como potencial advertencia de prediabetes.

En la literatura revisada no se ha encontrado ninguna investigación que utilice al FHTOA para identificar a personas con disglucemia (prediabetes y DM2) y resistencia a la insulina en sujetos con riesgo de DM2, con exceso de peso, ni en población general.

En Cuba, la alta prevalencia de los principales factores de riesgo cardiovascular -HTA (230,2 x 1000 habitantes) 21, sobrepeso (incremento del 44,8 al 56,1% de la población general) 22,23 y DM2 (66,9 x 1000 habitantes) 21- repercute en el incremento de la mortalidad por enfermedades cardiovasculares (2020-2021), con tasas de 267,3-384,9 x 100 000 habitantes, respectivamente 21,24. Asimismo, se ha observado un incremento de los factores de riesgo cardiovasculares en la población general 21-24, como también que la prevalencia de prediabetes y DM2 aumenta con la obesidad 25,26. En consecuencia, nos propusimos determinar la utilidad del fenotipo hipertensión-obesidad abdominal para detectar disglucemia y RI en personas con riesgo de DM2.

## MATERIAL Y MÉTODOS

Se llevó a cabo un estudio descriptivo, de tipo transversal, con 964 personas adultas (449 correspondieron al sexo femenino y 515 al masculino), que acudieron a consulta externa del Instituto de Endocrinología (INEN), a los cuales se les indicó la prueba de tolerancia oral a la glucosa (PTG-O) por sospecha de DM2, entre abril del 2008 y abril del 2013.

Los criterios de inclusión aplicados fueron: pacientes de ambos sexos que asistieron al laboratorio del INEN para realizarse una PTG-O por sospecha de DM2; y los de exclusión: presencia de embarazo o de cualquiera de las enfermedades endocrinas que se asocian con DM, padecer insuficiencia renal o hepática, tener antecedentes de enfermedad hematológica o maligna, ingestión de medicamentos con efecto hiperglucemiante o hipoglucemiante, y personas con indicación de PTG-O para estudio de causas diferentes al estudio de las disglucemias.

El fenotipo hipertensión-obesidad abdominal (FHTOA) 14 se definió como la presencia de presión sistólica ≥130 mmHg o presión diastólica ≥80 mmHg, o hipertensión tratada 14,27, más una CC >80 cm en mujeres y ≥90 cm en hombres 14,18.

Se consideró disglucemias a los diferentes estados de desregulación del metabolismo de la glucosa, comprendidos en las categorías de GAA, tolerancia a la glucosa alterada (TGA), prediabetes doble (combinación de GAA/TGA) y DM2, propuestos por la American Diabetes Association (ADA) 26. La investigación incluyó a individuos que presentaban al menos un factor de riesgo para DM2 26.

Como parte de la investigación, a cada paciente se le realizó un interrogatorio: edad, examen físico (peso, talla, tensión arterial, CC, color de la piel, búsqueda de Acantosis nigricans).

Los participantes fueron evaluados según peso, talla, CC e IMC (peso [kg]/talla [m^2^]). La medida de la CC se obtuvo con el empleo de una cinta métrica inextensible de menos de 1 cm de ancho, utilizando como punto de referencia el punto medio entre la última costilla y la cresta ilíaca y con el paciente en posición erecta, sin ropa en el abdomen y en espiración, en un plano horizontal al suelo. En los casos de abdómenes péndulos, la medición se hizo en decúbito supino, en el punto más prominente del abdomen, y se señaló en el modelo de recogida de datos.

La determinación de la TA a cada sujeto se midió por método auscultatorio, con esfigmomanómetro y adecuación del tamaño del manguito a la circunferencia braquial. Previamente, el sujeto estuvo sentado por 10 minutos. El procedimiento se llevó a cabo tres veces en el brazo derecho, con un intervalo de 5 minutos. El valor final de la TA correspondió al promedio de las tres mediciones obtenidas.

En todos los sujetos con sospecha de DM2 se determinó la presencia del FHTOA, según los criterios antes mencionados 14.

A cada persona se le hicieron las determinaciones en el momento de la extracción de la primera muestra sanguínea (basal), luego de aproximadamente 8-12 horas de ayuno.

A cada paciente se le indicó la prueba de tolerancia a la glucosa oral (PTG-O), con mediciones de glucemia en ayunas y a las 2 horas de la sobrecarga oral de glucosa, con 82,5 g de glucosa monohidratada 26.

La concentración de glucemia en ayunas y a las dos horas se midió en un analizador automático (Mindray BS-200E) por métodos enzimáticos.

Las determinaciones hormonales de insulinemia se hicieron utilizando kits diagnósticos proporcionados por el Centro de Isótopos (CENTIS), los cuales se utilizan de rutina en el laboratorio del INEN. La insulina se determinó por un método inmunoradiométrico (IRMA), comercializado por Izotop (Budapest-Hungría) [https://www.izotop.hu].

El índice de RI se calculó por el modelo homeostático de Mathews (HOMA-IR), siglas en inglés que hacen alusión a Homeostatic Model Assessment for Insulin Resistance. HOMA-IR = insulinemia en ayunas (µUI/mL) x glucemia en ayunas (mmol/L)/22,5 28.

### Análisis estadístico

Utilizamos promedios, desviación estándar y porcentajes. La comparación de promedios entre grupos se llevó a cabo con las pruebas t de Student o U de Mann-Whitney. La comparación de porcentajes se hizo con las pruebas X^2^ o exacta de Fisher entre las personas con y sin presencia del FHTOA. Se utilizó un intervalo de confianza (IC) del 95% y se consideró significancia estadística cuando p<0,0 5.

Se evaluó la relación entre las variables cuantitativas (edad, peso, IMC, glucemia en ayunas y a las dos horas durante la prueba de PTG-O, insulinemia, así como el índice HOMA-IR) y la CC y TA, utilizando el coeficiente de correlación de Pearson o Spearman, según corresponda.

Se determinó la efectividad del fenotipo hipertensión-obesidad abdominal para identificar a personas con disglucemia y resistencia a la insulina, mediante el cálculo de la sensibilidad, la especificidad, el valor predictivo positivo (VPP) y valor predictivo negativo (VPN).

### Consideraciones éticas

El proyecto fue aprobado por el Comité de Ética e Investigación del Instituto de Endocrinología, Universidad de Ciencias Médicas de la Habana (Cuba). La investigación se condujo de acuerdo con las guías propuestas en la Declaración de Helsinki. Se conservó la confidencialidad de los datos que se analizaron, y la identidad del participante se mantuvo en el anonimato.

## RESULTADOS

Se estudiaron 964 personas con algún factor de riesgo de DM2 (Tabla 1), entre las cuales predominaron los mayores de 45 años (57,2%), y la edad media fue de 46,9 años. Asimismo, predominó ligeramente el sexo masculino, con 515 hombres (53,4%). Hubo una mayoría de personas con piel blanca (634) (65,8%), seguidas de las que presentan piel mestiza o negra [198 (20,5%) y 132 (13,7%)], respectivamente. Se encontró la presencia de valores elevados de la circunferencia de la cintura (media de 95,3 cm), lo que sugiere la presencia de obesidad abdominal en varios sujetos. Además, se observó un incremento de los valores del IMC e índice HOMA-IR (media de 29,0 kg/m^2^ y 3,1, respectivamente) en muchos individuos.

**Tabla 1:** Características generales de los sujetos estudiados

Variables demográficas	N	%
Edad		
<45 años	413	42,8
≥45 años	551	57,2
Sexo		
Femenino	449	46,6
Masculino	515	53,4
Color de la piel		
Blanca	634	65,8
Negra	132	13,7
Mestiza	198	20,5
Acantosis nigricans	158	16,4
Variables clínicas y bioquímicas	Media	Desviación Estándar
Edad (años)	46,9	15,1
Peso (Kg)	79,6	20,5
Talla (cm)	165,2	9,2
Índice de masa corporal (kg/m^2^)	29,0	6,6
Circunferencia de la cintura (cm)	95,3	14,5
Tensión arterial sistólica (mm Hg)	119,7	15,5
Tensión arterial diastólica (mm Hg)	77,0	10,3
Glucosa en plasma ayunas (mmol/L)	5,2	1,5
Glucosa en plasma 2 horas (mmol/L)	6,3	2,8
Insulina (µU/mL)	13,0	1,7
Índice HOMA-IR	3,1	3,3

HOMA-IR: resistencia a la insulina por el índice HOMA-IR.

Se calculó la correlación de los componentes del FHTOA por separado (circunferencia de la cintura, tensión arterial sistólica y diastólica) con las variables estudiadas (edad, peso e IMC, glucosa en ayunas y 2 horas, insulina y HOMA-IR), y se encontró una correlación positiva con todas las variables (p<0,0001) (datos no mostrados).

En la Tabla 2 se muestran las características de las variables clínico-demográficas y bioquímicas en los individuos estudiados, según presencia o no del FHTOA. Encontramos mayor presencia del FHTOA en individuos mayores de 45 años (65,6%), en el sexo masculino (51,8%) y en las personas con color de piel negra (16,3%), en comparación con sujetos sin el fenotipo en cuestión (46,6, 40,1 y 10,5%, respectivamente). Además, se ha evidenciado una mayor frecuencia de Acantosis nigricans en las personas con el FHTOA (21,3%) (Tabla 2).

**Tabla 2:** Características demográficas, clínicas, antropométricas y bioquímicas de los sujetos estudiados según presencia del fenotipo hipertensión-obesidad abdominal

Variables demográficas y Acantosis nigricans	FHTOA N = 535 n (%)	No FHTOA N = 429 n (%)	p
Edad			
< 45 años	183 (34,3)	229 (53,4)	< 0,0001
≥45 años	351 (65,6)	200 (46,6)	
Sexo			
Femenino	258 (48,2)	257 (59,9)	< 0,0001
Masculino	277 (51,8)	172 (40,1)	
Color de la piel			
Blanca	338 (63,2)	296 (69,0)	
Negra	87 (16,3)	45 (10,5)	0,030
Mestiza	110 (20,6)	88 (20,5)	
Acantosis nigricans	114 (21,3)	44 (10,3)	< 0,0001
Variables clínicas y bioquímicas	Media ± DE	Media ± DE	p
Edad (años)	49,5 ± 14,0	43,7 ± 15,9	< 0,0001
Peso (Kg)	86,9 ± 21,0	70,4 ± 15,3	< 0,0001
Índice de masa corporal (kg/m^2^)	31,4 ± 6,5	26,1 ± 5,5	< 0,0001
Circunferencia de la cintura (cm)	106,4 ± 11,4	95,9 ± 12,1	< 0,0001
Glucosa en plasma ayunas (mmol/L)	5,4 ± 1,5	4,9 ± 1,4	< 0,0001
Glucosa en plasma 2 horas (mmol/L)	6,8 ± 2,9	5,7 ± 2,6	< 0,0001
Insulina (µU/mL)	14,2 ± 11,4	11,5 ± 11,9	< 0,0001
Índice homa-ir	3,5 ± 3,6	2,5 ± 2,8	< 0,0001

FHTOA: fenotipo hipertensión-obesidad abdominal. DE: desviación estándar; N: total de casos. n: número de casos con la condición. HOMA-IR: resistencia a la insulina por el índice HOMA-IR.

Se observó que los individuos con el FHTOA presentaban mayor edad y valores superiores de peso, IMC y perímetro de cadera, resultado estadísticamente significativo en relación con los individuos sin el FHTOA (Tabla 2). En la misma Tabla 2 observamos valores superiores en las concentraciones de glucemia en ayunas y dos horas post la PTG-O, insulinemia y en las cifras del índice HOMA-IR en las personas con el FHTOA, en comparación con aquellos sujetos sin el fenotipo (p<0,0001).

Al analizar la frecuencia de disglucemias y resistencia a la insulina en los sujetos, de acuerdo con la presencia o no del FHTOA, notamos que las personas con el FHTOA mostraron una mayor proporción de alteraciones del metabolismo de la glucosa (GAA, GAA/TGA y DM2) y resistencia a la insulina (RI) que aquellas personas sin el fenotipo. No obstante, el FHTOA no encuentra mayor frecuencia de sujetos con solo TGA en comparación con los individuos donde este fenotipo no está presente (Tabla 3).

**Tabla 3:** Frecuencias de personas detectadas con prediabetes, diabetes y resistencia a la insulina de acuerdo a la presencia o no del fenotipo hipertensión-obesidad abdominal

Condición	%	FHTOA	No FHTOA	Total
GAA	Sí	87 (65,9)^b^	45 (34,1)	132 (100)
	No	441 (53,6)	381 (46,3)	822 (100)
TGA	Sí	31 (59,6)	21 (40,4)	52 (100)
	No	495 (55,1)	404 (44,9)	899 (100)
GAA/TGA	Sí	55 (85,9)^a^	9 (14,1)	64 (100)
	No	471 (53,1)	416 (46,9)	887 (100)
DM2	Sí	62 (77,5)^a^	18 (22,5)	80 (100)
	No	464 (53,3)	407 (46,7)	871 (100)
Disglucemia	Sí	235 (71,6)^a^	93 (28,4)	328 (100)
	No	293 (46,8)	333 (53,2)	626 (100)
RI	Sí	221 (68,9)^a^	100 (31,1)	321 (100)
	No	264 (48,1)	285 (51,9)	549 (100)

FHTOA: fenotipo hipertensión-obesidad abdominal; GAA: glucosa alterada en ayunas. TGA: tolerancia a la glucosa alterada: GAA/TGA: prediabetes mixta. DM2: diabetes tipo 2; Disglucemia: GAA, TGA, GAA/TGA o DM2; RI: resistencia a la insulina; ^a^ p<0,0001; ^b^ p=0,009 vs. No FHTOA chi-cuadrado de Pearson

El FHTOA identificó la presencia de personas con disglucemias [GAA, TGA, prediabetes doble (GAA/TGA) y DM2] con una alta sensibilidad (71,6%) y una especificidad del 53,2%, así como un VPN del 78,2%. Es importante destacar que el FHTOA identifica mucho mejor a las personas con presencia de prediabetes doble (GAA/TGA) y DM2, pues muestra sensibilidades mucho más altas (85,9 y 77,5%, respectivamente), así como VPN elevados (97,9 y 95,8%, respectivamente) (Tabla 4). La sensibilidad, la especificidad y el VPN del FHTOA para detectar personas con resistencia a la insulina fue de 68,9, 51,9 y 74,0%, respectivamente (Tabla 4).

**Tabla 4:** Precisión de diagnóstico del fenotipo hipertensión-obesidad abdominal para identificar a personas con disglucemias y resistencia a la insulina

Condición		FHTOA		
	Sensibilidad% IC 95%	Especificidad% IC 95%	VPP% IC 95%	VPN% IC (95%)
GAA	65,9 (57,4-74,4)	46,4 (42,9-49,9)	16,5 (13,2-19,7)	89,4 (86,4-92,5)
TGA	59,6 (45,3-73,9)	44,9 (41,6-48,3)	5,9 (3,8-8,0)	95,1 (92,9-97,2)
GAA/TGA	85,9 (76,6-95,2)	46,9 (43,6-50,2)	10,5 (7,8-13,2)	97,9 (96,4-99,4)
DM2	77,5 (67,7-87,3)	46,7 (43,7-50,1)	11,8 (8,9-14,6)	95,8 (93,7-97,8)
Disglucemia	71,6 (66,6-76,7)	53,2 (49,2-57,2)	44,5 (40,2-48,8)	78,2 (74,1-82,2)
RI	68,9 (63,6-74,1)	51,9 (47,6-56,2)	45,6 (41,0-50,1)	74,0 (69,5-78,5)

IC: intervalos de confianza; FHTOA: fenotipo hipertensión-obesidad abdominal; GAA: glucosa alterada en ayunas; TGA: tolerancia a la glucosa alterada; DM2: diabetes tipo 2; Disglucemia: GAA, TGA, GAA/TGA o DM2; RI: resistencia a la insulina; VPP: valor predictivo positivo; VPN: valor predictivo negativo.

## DISCUSIÓN

La obesidad se considera un factor de riesgo importante para la enfermedad cardiovascular y se asocia con el desarrollo de hiperinsulinemia, resistencia a la insulina, intolerancia a la glucosa, hipertensión arterial, dislipidemias, hiperuricemia y el llamado síndrome metabólico 2-5,8,9,16,19,20,25,29-31.

Diferentes estudios han mostrado que el FHTOA es un marcador sencillo y útil para identificar a los pacientes con obesidad abdominal que tienen alterado el perfil metabólico, sin necesidad de utilizar pruebas adicionales 13,14. La determinación de la hipertensión arterial y de la circunferencia de la cintura es un procedimiento fácilmente accesible y económico, y la medición de estas dos variables carece de costo 13,14.

Los resultados del presente estudio muestran que la frecuencia del FHTOA en personas con riesgo de padecer DM2 fue del 55,5%, es superior a la encontrada por Nita *et al.* en población general (43,3%) 13. Este es un resultado esperado, pues la investigación se llevó a cabo en personas con riesgo ya identificado de diabetes. La HTA está asociada con la obesidad abdominal (adiposidad central) 5,8,17,20. Cabrera-Rode *et al.*^14^ encontraron una frecuencia más alta del FHTOA en adultos con exceso de peso (76,9%).

La alta proporción de hombres con el FHTOA en este estudio debe ser el resultado de que la frecuencia de la HTA fue superior en los hombres en comparación con las mujeres. Datos similares con relación a la alta frecuencia de HTA en hombres se han encontrado en algunos estudios en Cuba 32,33 y Latinoamérica 34.

Los datos de la presente investigación muestran que la frecuencia del FHTOA fue superior en las personas de color de piel negra, en comparación con los individuos de color de piel blanca o mestizo. Cabrera-Rode *et al.*^14^, en una investigación anterior sobre el fenotipo en cuestión, encontraron una tendencia a la asociación con el color de piel negra o mestizo (p=0,057). Orduñez no encontró diferencias en la frecuencia de hipertensión por el color de la piel en Cuba 35. Como se aprecia, existen resultados controversiales que probablemente estén en correspondencia con la población objeto de estudio.

Se observó que en las personas con Acantosis nigricans (AN), el FHTOA era más frecuente, lo cual afirma la asociación de la AN con la hipertensión 36.

Esta investigación encontró una correlación positiva de la tensión arterial y la circunferencia de la cintura (componentes del FHTOA) con la resistencia a la insulina, la insulinemia y disglucemia. Simultáneamente, al analizar los valores absolutos de las variables antes mencionadas (HOMA-IR, insulinemia, glucemia en ayunas y a las 2 horas) vemos que las personas con el FHTOA presentaron cifras más elevadas en comparación con aquellas personas sin el fenotipo. Diversos autores han reafirmado la correlación de la tensión arterial y la circunferencia de la cintura con las variables anteriormente mencionadas 8,16-19.

Otro resultado interesante fue el hecho de que el FHTOA detectó mayor proporción de sujetos con disglucemia y resistencia a la insulina, lo que resalta que la presencia de este binomio pudiera ser una señal para la realización de exámenes de laboratorio en sujetos que verdaderamente lo requieran (insulinemia, glucemia en ayunas y a las 2 horas), que identifiquen trastornos del metabolismo de la glucosa.

Diferentes estudios apoyan la asociación de la hipertensión y la obesidad abdominal con la prediabetes y la diabetes, así como con la hiperinsulinemia y RI 8,14,17-20,37,38.

Las evidencias de esta investigación consolidaron aún más las relaciones entre los componentes del FHTOA con la resistencia a la insulina y las alteraciones del metabolismo de la glucosa. Es importante destacar que dentro de la disglucemia, el FHTOA selecciona significativamente a las personas fundamentalmente con prediabetes doble (incluye a la GAA/TGA al mismo tiempo) y DM2, además de la GAA. Esto indica que utilizar al FHTOA en la práctica clínica simplificaría la detección de personas con GAA, GAA/TGA y DM2.

Cabrera-Rode *et al.*^14^ hallaron que las personas con el FHTOA presentaron una frecuencia superior de glucosa alterada en ayunas (GAA), en comparación con aquellos sin el fenotipo. Es decir, que del total de personas con GAA (n = 74), el FHTOA mostró una elevada proporción de personas con esa condición [86,5% (64/74)] 14. En contraste, en esta investigación, el FHTOA detectó al 65,9% (87/132), 85,9% (55/64), 77,5% (62/80) y 68,9% (221/321) del total de personas con GAA, GAA/TGA, DM2 y RI, respectivamente.

La alta frecuencia de GAA, prediabetes doble, DM2 y RI en el FHTOA, del total de casos con las condiciones antes mencionadas, se debe a que estudiamos personas con riesgo de *diabetes mellitus* tipo 2. Estos resultados revelan al FHTOA como potencial advertencia de prediabetes (GAA y GAA/TGA), DM2 y RI. Por otra parte, en la literatura revisada no encontramos otra información sobre la asociación del binomio hipertensión-obesidad abdominal con la presencia de resistencia a la insulina y disglucemia.

Este estudio mostró, además, la alta sensibilidad del FHTOA, en la detección de personas con prediabetes doble y DM2 (85,9 y 77,5%, respectivamente), así como VPN altos para el mismo propósito. Los valores altos de sensibilidad y de VPN para el doble orientan a que la asociación del binomio hipertensión-obesidad abdominal se utilice como herramienta para la detección de personas con prediabetes doble y DM2.

Notamos que la presencia del FHTOA mostró una cantidad de falsos positivos (especificidad del 46,9 y 46,7%) y reveló una alta probabilidad para descartar realmente a los sujetos que no tengan prediabetes doble y DM2 (VPN del 97,9 y 95,8%, respectivamente). Al contrario, el FHTOA exhibió baja sensibilidad (59,6%) para identificar personas con TGA y un alto VPN (95,1%), por tanto, el no presentar este fenotipo descarta realmente a las personas con TGA. No obstante, el binomio hipertensión-obesidad abdominal mostró una sensibilidad no tan alta (65,9%) y ostentó VPN casi del 90%, por tal razón tiene una probabilidad para descartar realmente los sujetos que no tengan GAA.

Nuestro estudio confirma que el FHTOA (alta sensibilidad) es útil para identificar o descartar (alto VPN) a las personas con disglucemias, pues el no presentar el FHTOA descarta que probablemente la persona tenga disglucemia. Esto quiere decir que la identificación de sujetos con el FHTOA da muy pocos falsos negativos, pero si un número elevado de falsos positivos.

Cabrera Rode *et al.*^14^ encontraron una alta sensibilidad (90,5%) del FHTOA para identificar a personas con SM, así como VPN altos para el mismo propósito en personas con exceso de peso. No obstante, Nita *et al.*^13^ encontraron, en población general, que el FHTOA presentó también una alta sensibilidad (80,4%) para identificar a personas con SM. Lo mencionado indica que en esta investigación se evidencia que el FHTOA pudiera identificar a personas con prediabetes doble y DM2 con similar sensibilidad para detectar SM. Sin embargo, la no presencia del FHTOA descarta (alto VPN) que probablemente la persona tenga disglucemias y resistencia a la insulina.

El FHTOA revela muy pocos falsos negativos, pero al mismo tiempo un número elevado de falsos positivos para identificar a personas con disglucemias o resistencia a la insulina. Este último razonamiento es lógico, ya que podemos encontrar personas que al momento del estudio presentan solo el binomio hipertensión-obesidad abdominal y aún no ha aparecido la resistencia a la insulina o alguna alteración del metabolismo de la glucosa. Por lo anterior, urge tratar intensivamente la hipertensión y la obesidad en las personas con el FHTOA, para evitar la progresión hacia trastornos metabólicos.

La limitación principal de este estudio radica en que, al haber incluido a personas con riesgo de *diabetes mellitus* tipo 2 que acudieron de manera consecutiva a un proyecto de investigación, hace que sea un muestreo no probabilístico. No obstante, la importancia del estudio residen en que, que hasta donde sabemos, no existen estudios en los cuales se compare la sensibilidad, la especificidad y los valores predictivos entre el FHTOA y la resistencia a la insulina, así como las alteraciones metabólicas antes referidas para identificar a personas con disglucemia.

En síntesis, la combinación de los componentes HTA y la obesidad abdominal, es capaz de detectar una gran cantidad de personas con alteraciones del metabolismo de la glucosa y resistencia a la insulina, en comparación con los sujetos sin el fenotipo en cuestión. Por tanto, se recomienda que todos los médicos incluyan la determinación de la glucosa plasmática durante una PTG-O o insulinemia en sus pruebas solicitadas de forma rutinaria para las personas con el FHTOA. De lo contrario, se espera que el manejo de la presión arterial elevada y la obesidad abdominal no sea el óptimo necesario para la prevención de las alteraciones del metabolismo de la glucosa 8,37,38.

El presente estudio reitera la importancia de la utilidad clínica del FHTOA y la posibilidad que ofrece de ser utilizado en grandes poblaciones, incluida la atención primaria de salud.

En conclusión, el fenotipo hipertensión-obesidad abdominal es una opción sencilla, útil para identificar a personas con disglucemia y resistencia a la insulina.

La aplicación de este binomio es económica, pues no utiliza mediciones de laboratorio. Por ende, es recomendable realizar otros estudios semejantes en población general que sustenten estos resultados ♣

## References

[1] Müller TD, Blüher M, Tschbp MH, DiMarchi RD (2022). Anti-obesity drug disco-very: advances and challenges. Nat Rev Drug Discov.

[2] Zhang P, Sun X, Jin H, Zhang F-L, Guo Z-N, Yang Y (2019). Association be-tween obesity type and common vascular and metabolic diseases: A cross-sectional study. Front Endocrinol (Lausanne).

[3] Oussaada SM, van Galen KA, Cooiman MI, Kleinendorst L, Hazebroek EJ, van Haelst MM (2019). The pathogenesis of obesity. Metabolism.

[4] Choi D, Choi S, Son JS, Oh SW, Park SM (2019). Impact of discrepancies in general and abdominal obesity on major adverse cardiac events. J Am Heart Assoc.

[5] Wang Y, Howard AG, Adair LS, Wang H, Avery CL, Gordon-Larsen P (2020). Waist circumference change is associated with blood pressure change independent of BMI change. Obesity (Silver Spring).

[6] Coutinho T, Goel K, Correa de Sá D, Carter RE, Hodge DO, Kragelund C (2013). Combining body mass index with measures of central obesity in the assessment of mortality in subjects with coronary disease: role of "normal weight central obesity". J Am Coll Cardiol.

[7] Kim HY, Kim JK, Shin GG, Han JA, Kim JW (2019). Association between abdominal obesity and cardiovascular risk factors in adults with normal body mass index: Based on the sixth Korea national health and nutrition examination survey. J Obes Metab Syndr.

[8] Sangrós FJ, Torrecilla J, Giráldez-García C, Carrillo L, Mancera J, Mur T (2018). Association of general and abdominal obesity with hypertension, dyslipidemia and prediabetes in the PREDAPS Study. Rev Esp Cardiol (Engl Ed).

[9] Diéguez-Martínez M, Miguel-Soca PE, Rodríguez-Hernández R, López-Báster J, Ponce-de León D (2017). Prevalencia de obesidad abdominal y factores de riesgo cardiovascular asociados en adultos jóvenes. Rev Cubana Salud Pública.

[10] Ribeiro FB, de Cássia da Silva C, Vasques ACJ, Zambon MP, De Bernardi Rodrigues AM, Camilo DF (2015). Hypertriglyceridemic waist phenotype indicates insulin resistance in adolescents: validation study front hyperglycemic clamp-Brazilian Metabolic Syndrome Study (BRAMS). Diabetol Metab Syndr.

[11] Díaz-Santana MV, Suárez-Pérez EL, Ortiz-Martínez AP, Guzmán-Serrano M, Pérez-Cardona CM (2016). Association between the hypertriglyceridemic waist phenotype, prediabetes, and diabetes mellitus among adults in Puerto Rico. J Immigr Minor Health.

[12] Chen S, Guo X, Yu S, Yang H, Sun G, Li Z (2016). Hypertriglyceridemic waist phenotype and metabolic abnormalities in hypertensive adults: A STROBE compliant study. Medicine (Baltimore).

[13] Nita C, Hancu N, Rusu A, Bala C, Roman G (2009). Hypertensive waist: first step of the screening for metabolic syndrome. Metab Syndr Relat Disord.

[14] Cabrera-Rode E, Borja Coronel AC, Montes de Oca-Somoano R, Rodríguez-Acosta J, Cubas-Dueñas I, Arnold-Domínguez Y (2020). Utilidad del fenotipo hipertensión-obesidad abdominal para identificar personas con síndrome metabólico. Revista ALAD.

[15] Parlá-Sardiñas J, Cabrera-Rode E, Rodríguez-Acosta J, Cubas-Dueñas I, Arnold-Domínguez Y, Hernández-Rodríguez J (2020). Utilidad del fenotipo hipertensión-obesidad abdominal para identificar personas con riesgo cardiovascular global moderado y alto. Rev Cubana Endocrinol.

[16] Cheng YH, Tsao YC, Tzeng IS, Chuang HH, Li WC, Tung TH (2017). Body mass index and waist circumference are better predictors of insulin resistance than total body fat percentage in middle-aged and elderly Taiwanese. Medicine (Baltimore).

[17] Darsini D, Hamidah H, Notobroto HB, Cahyono EA (2020). Health risks associated with high waist circumference: A systematic review. J Public Health Res.

[18] Díaz O, Hernández J, Domínguez E, Martínez I, Bosch Y, del Busto A (2017). Valor de corte de la circunferencia de la cintura como predictor de disglucemia. Rev Cubana Endocrinol.

[19] Hu FB, Stampfer MJ (2005). Insulin resistance and hypertension: the chic-ken-egg question revisited. Circulation.

[20] Zhang T, Zhang H, Li S, Li Y, Liu Y, Fernandez C (2016). Impact of adiposity on incident hypertension is modified by insulin resistance in adults: Longitudinal observation from the Bogalusa Heart Study. Hypertension.

[21] Ministerio de Salud Pública. Dirección nacional de estadísticas y registros médicos (2022). Anuario estadístico de salud 2021.

[22] Bonet M, Varona P, Chang M, García RG, Suarez R, Arcía N (2014). III Encuesta nacional de factores de riesgo y actividades preventivas de enfermedades no trasmisibles. Cuba 2010-2011.

[23] Díaz-Sánchez ME, Maldonado G, Suárez-Medina R, Varona P (2022). Nuevos datos sobre el sobrepeso y la obesidad en Cuba.

[24] Ministerio de Salud Pública. Dirección nacional de estadísticas y registros médicos (2021). Anuario estadístico de salud 2020.

[25] Ortega MA, Fraile-Martínez O, Naya I, García-Honduvilla N, Álvarez-Mon M, Buján J (2020). Type 2 diabetes mellitus associated with obesity (diabesity). The central role of gut microbiota and its translational applications. Nutrients.

[26] ElSayed NA, Aleppo G, Aroda VR, Bannuru RR, Brown FM (2023). American Diabetes Association. 2. Classification and diagnosis of diabetes: standards of care in Diabetes-2023. Diab Care.

[27] helton PK, Carey RM, Aronow WS, Casey DE, Collins KJ, Dennison-Himmelfarb C (2018). 2017 ACC/AHA/AAPA/ABC/ACPM/AGS/APhA/ASH/ASPC/NMA/PCNA guideline for the prevention, detection, evaluation, and management of high blood pressure in adults: Executive summary: A report of the American college of cardiology/American heart association task force on clinical practice guidelines. Hypertension.

[28] Arranz-Calzado C, González-Suárez RM, Álvarez-Álvarez A, Rodríguez-Pendás B, Reyes-Durán A (2010). Criterios de referencia para los indicadores de secreción de insulina y de los parámetros lipídicos en una población mixta hospitalaria. Rev Cubana Endocrinol.

[29] Inoue Y, Qin B, Poti J, Sokol R, Gordon-Larsen P (2018). Epidemiology of obesity in adults: Latest trends. Curr Obes Rep.

[30] Qing L, Wei R, Chan L, Xiaoya Z, Xin X (2017). Sensitivity of various body indices and visceral adiposity index in predicting metabolic syndrome among Chinese patients with adult growth hormone deficiency. J Endocrinol Invest.

[31] Zeng J, Lawrence WR, Yang J, Tian J, Li C, Lian W (2021). Association between serum uric acid and obesity in Chinese adults: a 9-year longitudinal data analysis. BMJ Open.

[32] Delgado-Acosta H, Lastre-Navarro K, Valdés-Gómez M, Benet-Rodríguez M, Morejón-Giraldoni AF, Zerquera-Rodríguez J (2015). Prevalencia de hipertensión arterial en el Área I del municipio Cienfuegos. Segunda medición de la iniciativa CARMEN: cifras alarmantes. Rev Finlay.

[33] Miguel-Soca PE, Rivas-Estévez M, Sarmiento-Teruel Y, Mariño-Soler AL, Marrero-Hidalgo M, Mosqueda-Batista L (2016). Prevalence of metabolic syndrome risk factors in adults in Holguín, Cuba (2004-2013). MEDICC Rev.

[34] Wong-McClure RA, Gregg EW, Barceló A, Lee K, Abarca-Gómez L, Sanabria-López L (2015). Prevalence of metabolic syndrome in Central America: a cross-sectional population-based study. Rev Panam Salud Publica.

[35] Ordúñez P, Kaufman JS, Benet M, Morejon A, Silva LC, Shoham DA (2013). Blacks and whites in the Cuba have equal prevalence of hypertension: confirmation from a new population survey. BMC Public Health.

[36] Montoya Rodríguez MA (2020). Acantosis nigricans como factor asociado a hipertensión arterial en pacientes del Hospital Belén de Trujillo.

[37] Da Silva AA, do Carmo JM, Li X, Wang Z, Mouton AJ, Hall JE (2020). Role of hyperinsulinemia and insulin resistance in hypertension: Metabolic syndrome revisited. Can J Cardiol.

[38] Mancusi C, Izzo R, di Gioia G, Losi MA, Barbato E, Morisco C (2020). Insulin resistance the hinge between hypertension and type 2 diabetes. High Blood Press Cardiovasc Prev.

